# Surgical Management of Congenital Pulmonary Airway Malformations (CPAM) in an Infant and a Toddler: Case Report Depicting Two Distinct Surgical Techniques With Successful Outcomes

**DOI:** 10.7759/cureus.53526

**Published:** 2024-02-03

**Authors:** Vishal V Bhende, Tanishq S Sharma, Mathangi Krishnakumar, Deepali M Shah, Rajesh N Pankhaniya, Zalak N Parmar, Alpa M Patel, Dharmendra B Parmar, Amit Kumar, Kartik B Dhami, Sohilkhan R Pathan, Ashwin S Sharma, Vrajana J Parikh, Haryax V Pathak, Rushi B Barot, Dimple B Shah, Swati M Kamani, Nili J Mehta, Gaurav D Bhoraniya, Roshni A Purswani

**Affiliations:** 1 Pediatric Cardiac Surgery, Bhanubhai and Madhuben Patel Cardiac Centre, Shree Krishna Hospital, Bhaikaka University, Karamsad, IND; 2 Anesthesiology, St. John's Medical College Hospital, Bengaluru, IND; 3 Pediatrics, Pramukh Swami Medical College, Shree Krishna Hospital, Bhaikaka University, Karamsad, IND; 4 Pathology, Pramukh Swami Medical College, Shree Krishna Hospital, Bhaikaka University, Karamsad, IND; 5 Anesthesiology, Pramukh Swami Medical College, Shree Krishna Hospital, Bhaikaka University, Karamsad, IND; 6 Radiodiagnosis, Pramukh Swami Medical College, Shree Krishna Hospital, Bhaikaka University, Karamsad, IND; 7 Pediatric Cardiac Intensive Care, Bhanubhai and Madhuben Patel Cardiac Centre, Shree Krishna Hospital, Bhaikaka University, Karamsad, IND; 8 Cardiac Anesthesiology, Bhanubhai and Madhuben Patel Cardiac Centre, Shree Krishna Hospital, Bhaikaka University, Karamsad, IND; 9 Clinical Research Services, Bhanubhai and Madhuben Patel Cardiac Centre, Shree Krishna Hospital, Bhaikaka University, Karamsad, IND; 10 Internal Medicine, Gujarat Cancer Society Medical College, Hospital and Research Centre, Ahmedabad, IND; 11 Pediatric Critical Care, Bhanubhai and Madhuben Patel Cardiac Centre, Shree Krishna Hospital, Bhaikaka University, Karamsad, IND; 12 Surgery, Pramukh Swami Medical College, Shree Krishna Hospital, Bhaikaka University, Karamsad, IND

**Keywords:** lung-sparing resection, congenital cystic lung, toddler, infant, conservative vs surgical management, surgical management, wedge resection, segmental resection, congenital pulmonary airway malformation, congenital cystic adenomatoid malfomation

## Abstract

Congenital pulmonary airway malformations (CPAM) compose the major part of congenital lung malformations (CLM) and have traditionally been treated by pulmonary lobectomy. In terms of surgical strategy, lobectomy has conventionally been the preferred treatment for CPAM localized to a single lobe. More recently, alternative approaches including lung-sparing resections (LSR), such as wedge or non-anatomic resections and segmentectomy, have been suggested. In asymptomatic CPAM early surgical resection is often shown to reduce infection and malignancy development.

We describe two patients who were diagnosed with CPAM when being evaluated for respiratory tract infection. Patient 1 (P1) was a two-month-old infant weighing 4 kg with glucose-6-phosphate dehydrogenase (G6PD) deficiency and Patient 2 (P2) was a toddler aged one year, nine months weighing 9 kg. P1 underwent LSR for the CPAM diagnosed in the left upper lobe of the lung with conventional mechanical ventilation whilst right upper lobectomy was performed in P2 using one/single lung ventilation. In both cases, LSR and right upper lobectomy led to an uneventful postoperative recovery with no complications reported.

## Introduction

Congenital pulmonary airway malformations (CPAM) constitute the core of congenital lung malformations (CLM). In this condition, there is excessive proliferation of the respiratory tree leading to an abnormal bulk of tissue and resulting in anomalous alveoli development [1]. The actual incidence remains uncertain, with prior approximations varying from 1:11,000 to 1:35,000 live births, but current indications suggest it is approximately 1:7,200. Studies have indicated male predominance in lesions appearing during early infancy. Advancements in prenatal ultrasound methodologies, increased accessibility to prenatal therapies and interventions for fetal issues, and improvements in neonatal intensive care have all contributed to progress in perinatal healthcare and possibly to the increase in the reported incidences [2].

While the exact origin of CPAM remains uncertain, it is believed to stem from hamartomatous malformation and excessive pulmonary tissue proliferation at different locations [3]. The Stocker’s classification uses the diameter of the cyst and location to categorize CPAM into five main types (Type 0 to Type 4) according to histopathological criteria [4].

CPAM, originally referred to as congenital cystic adenoid malformation (CCAM), was first outlined as a distinct medical condition by Ch’in and Tang in 1949. Initially categorized as three types in 1977 [4], Stocker et al. expanded the classification to five types in 2002 and subsequently coined the term CPAM. Roughly 80% to 85% of instances are commonly identified during the initial two years of an individual's life, with rare occurrences in adults. CPAM usually affects a single lobe and frequently manifests with symptoms such as infection or abscess. While resection is the established treatment for symptomatic CPAM, the approach to managing asymptomatic cases remains a subject of debate. Despite considering the associated risks of morbidity and mortality with surgery, several studies advocate for early surgical intervention to prevent potential complications, such as spontaneous pneumothorax, recurrent infections, or occurence of lung tumors [5]. The objective of this investigation is to present surgical findings regarding CPAM in an infant and a toddler and to assess the most suitable timing for surgical treatment.

## Case presentation

The study received approval from the Institutional Ethics Committee (IEC-2) at H.M. Patel Centre for Medical Care and Education, Anand, Gujarat (Approval No. IEC/BU/2023/Cr. 43/365/2023 dated 04/12/2023). The authors affirm that this report contains no personal information enabling patient identification. Given that the subjects are minors, explicit written consent was acquired from their parents.

Case 1 (P₁)

**Figure 1:**
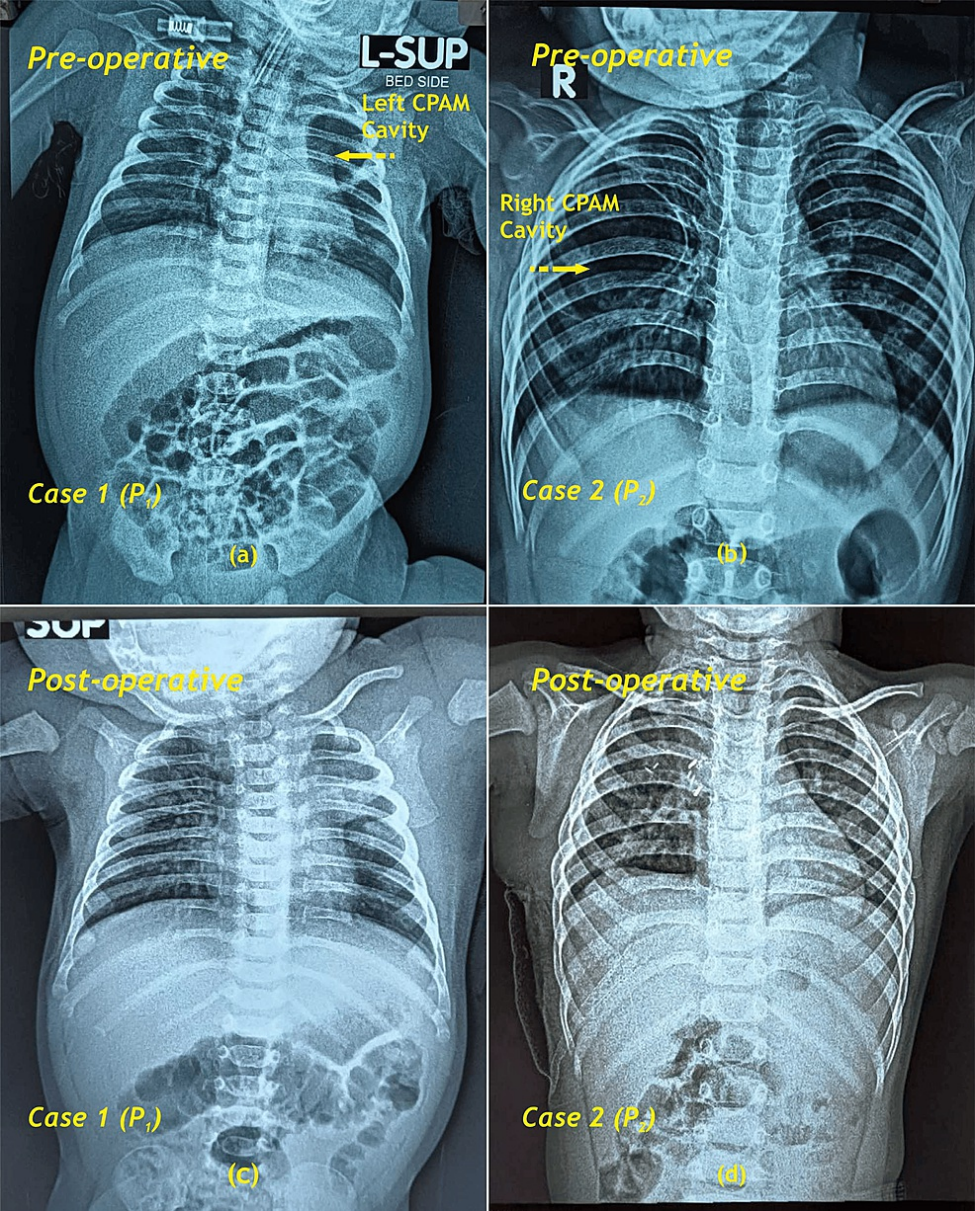
CXR pre-operative: (a) P1 showing CPAM cavity in left upper lobe of lung, (b) P2 showing CPAM cavity in right upper lobe of lung, (c) & (d) CXR postoperative CXR - Chest X-ray; CPAM - Congenital pulmonary airway malformation; P - Patient Image Credit: Dr. Vishal V. Bhende

P1 was a two-month-old boy weighing 4 kg born late preterm via vaginal delivery who had neonatal jaundice and glucose-6-phosphate dehydrogenase (G6PD) deficiency. He received phototherapy for neonatal jaundice. He presented at our pediatric outpatient clinic post-discharge with complaints of ear discharge and fever and was in due course diagnosed with late-onset sepsis with infection of the lung. The first chest X-ray (CXR) suggested haziness in left upper lobe. However, serial CXRs showed a cavitary lesion in the left upper lobe (Figure 1).

As the patient had persistent requirement for ventilatory support and respiratory distress, high resolution contrast-enhanced computed tomography chest (HRCECT) was done which revealed presence of Type I CPAM.

Case 2 (P₂) 

**Figure 2:**
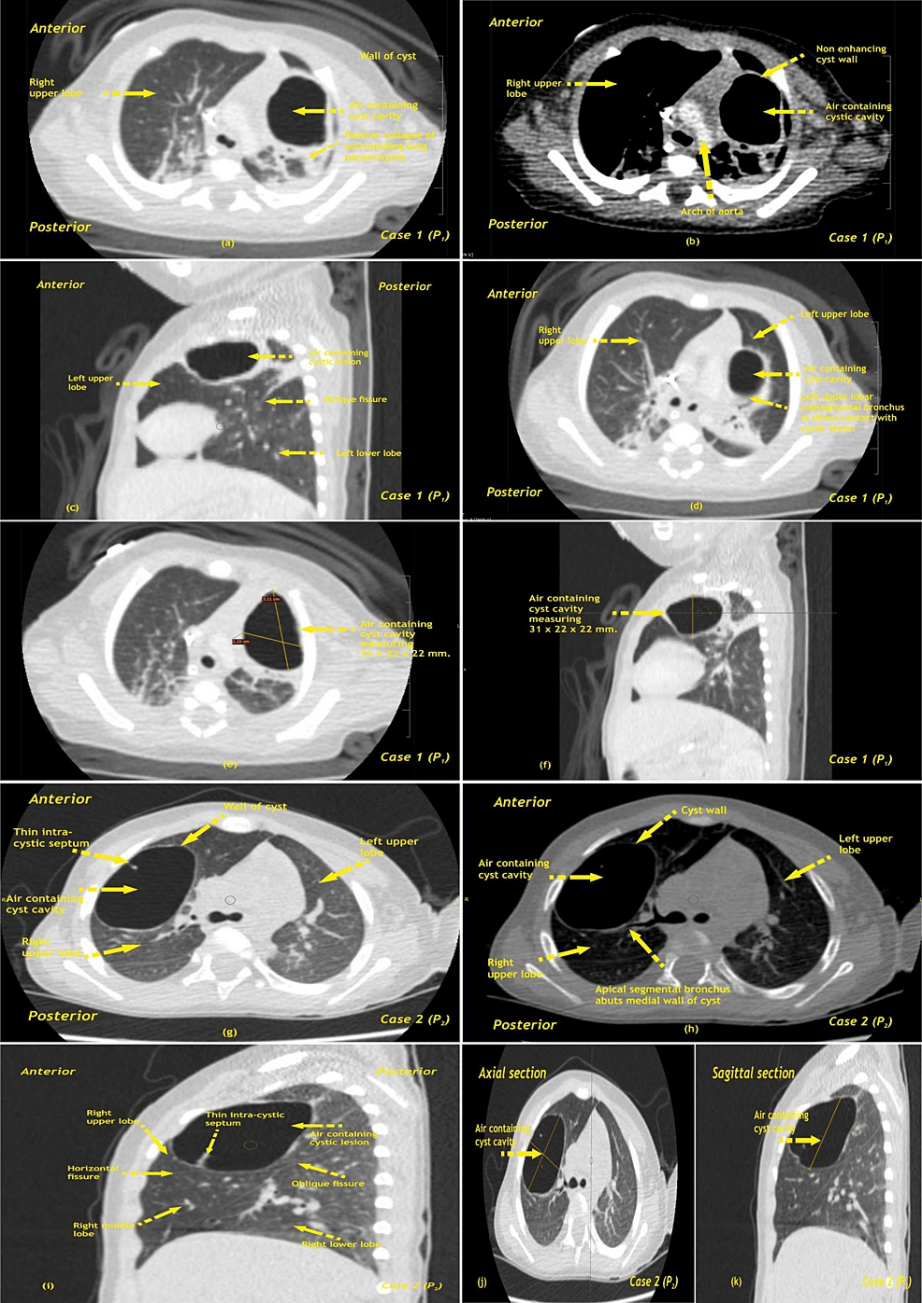
Case 1 (P1) (a) CECT scan of chest axial section in lung window (section 1) at the level of upper lobe showing a well-defined, thin-walled air containing cystic cavity lesion with surrounding lung parenchyma shows changes of passive collapse in left lung upper lobe. (b) CECT of section 1 in mediastinal window at level of upper lobe showing well-defined air-containing cavernous lesion with thin wall on CECT scan. (c) CECT of chest sagittal section in lung window showing air containing well-defined cystic lesion noted in left upper lobe with adjacent areas of collapse and left oblique fissure which separate left upper and lower lobe. (d) CECT of (section 1) in lung window at level of upper lobe showing a well-defined air-containing cavernous lesion in left upper lobe with upper lobar subsegmental bronchus appear in direct contact with cystic lesion and adjacent areas of passive collapse. (e) CECT of (section 1) showing well-defined air-containing cystic lesion in upper lobe of left lung measuring approximately 31 x 22 x 21 mm suggests CPAM (Type I) more likely. (f) CECT of chest sagittal section showing well-defined air-containing cystic lesion in upper lobe of left lung measuring approximately 31 x 22 x 21 mm suggests CPAM (Type I) more likely. Case 2 (P2) (g) HRCT scan of (section 1) at level of upper lobe showing a big, well-defined, oval, thin-walled, air-containing cystic cavity lesion in anterior segment of right upper lobe. There is thin oblique internal septation noted in antero-inferior portion of cavity. (h) HRCT scan of (section 1) in mediastinal window at level of upper lobe showing a big, well-defined, oval, thin walled, air containing cystic cavity lesion in anterior segment of right upper lobe. Apical segmental bronchus abuts medial wall of cystic lesion. (i) HRCT scan of chest sagittal section in lung window at right lung showing a big, well-defined, oval, thin-walled, air-containing cystic cavity lesion in anterior segment of right upper lobe. There is thin oblique internal septation noted in antero-inferior portion of cavity. The cystic lesion also abuts posteriorly to oblique fissure and inferiorly to horizontal fissure. (j & k) HRCT scan of chest axial and sagittal sections showing big, well-defined, oval, thin-walled, air-containing cystic lesion in anterior segment of right upper lobe measuring approximately 52.5 x 36.6 x 41.7 mm (AP X TX X SI) suggests congenital pulmonary airway malformation (Type I) more likely. CECT - Contrast-enhanced computed tomography; HRCT - High-resolution computed tomography; CPAM - Congenital pulmonary airway malformation; AP - Antero-posterior; TX - Transverse; SI - Supero-inferior; P - Patient Image Credit: Dr. Dharmendra B. Parmar

The second patient is a one-year, nine-month-old toddler weighing 9 kg born full term via vaginal delivery and having history of neonatal jaundice requiring phototherapy and presented with respiratory tract infection and slowly progressive protrusion of lower part of sternum. CXR was done to rule out any abnormality and revealed a large radiolucent shadow in the right lung. Further evaluation via imaging showed Type I CPAM in the right upper lobe of the lung (Figure 2).

Both patients were optimized for surgery by 2D echocardiography which depicted normal heart structure. Besides routine laboratory investigations, the results of SARS-CoV-2 coronavirus disease 2019 (COVID-19) analyses were negative (Table 1).

**Table 1:** Patient Characteristics P - Patient

Sr. No.	Parameters	Case 1 (P_1_)	Case 2 (P_2_)
1	Age	2 months	1 year, 9 months
2	Sex	Male	Male
3	Prenatal Diagnosis	No	No
4	Symptomatic	Yes	No
5	Lobar Involvement	Left upper lobe	Right upper lobe

Anesthesia protocol

**Figure 3:**
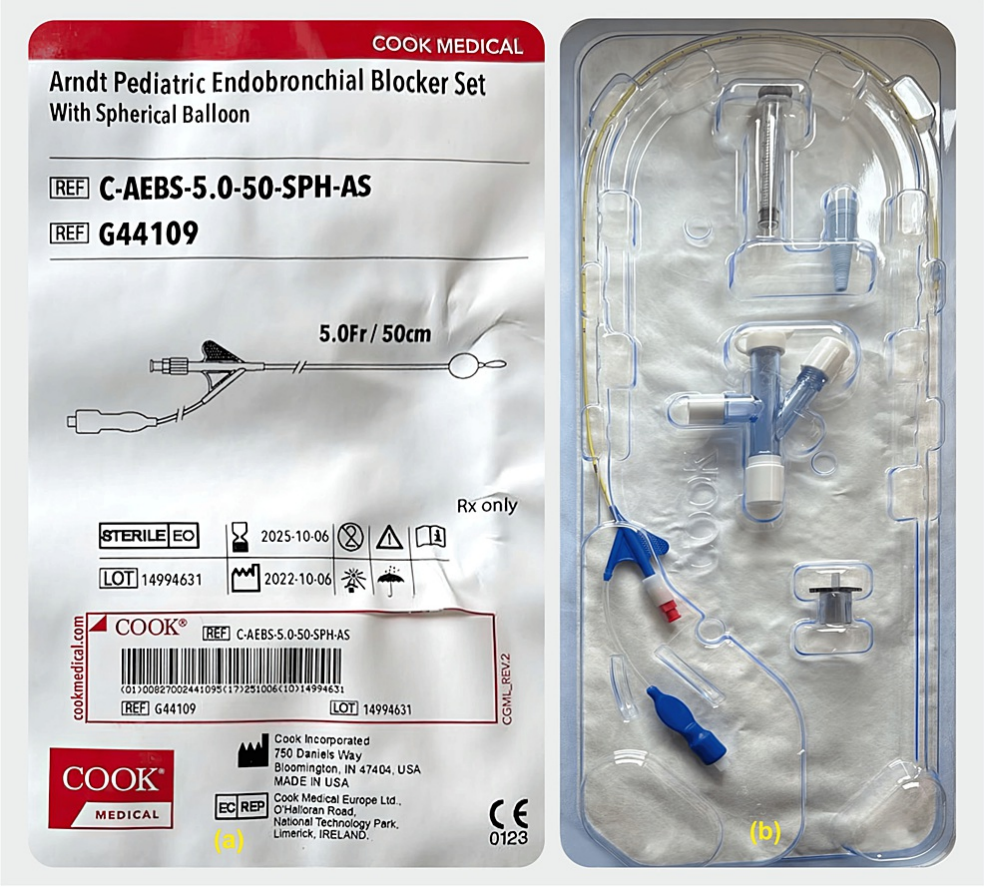
Arndt Pediatric Endobronchial Blocker 5 Fr. (Cook Medical, Bloomington, IN, USA) with internal assembly (a & b) Image Credit: Dr. Vishal V. Bhende

The induction of anesthesia is very crucial during the surgery of CLM. In P1, a single lumen endotracheal tube was used for tracheal intubation. In P2 one-lung ventilation (OLV) using Arndt Pediatric Endobronchial Blocker 5 Fr. (Cook Medical, Bloomington, IN, USA) was adopted (Figure 3).

Surgical technique

**Figure 4:**
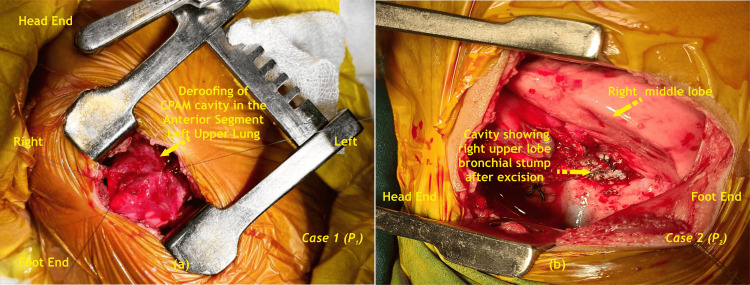
(a) Left limited thoracotomy exposure in Case 1 (b) Right postero-lateral thoracotomy exposure in Case 2 CPAM - Congenital pulmonary airway malformation; P - Patient Image Credit: Dr. Vishal V. Bhende

**Figure 5:**
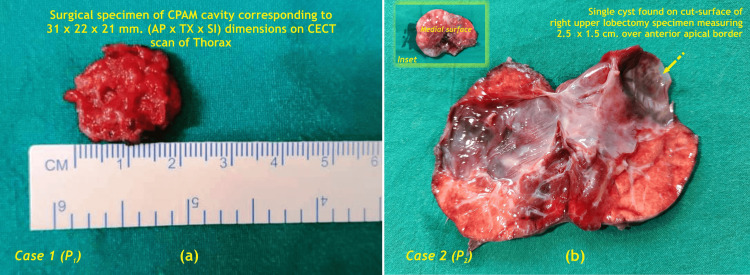
Surgical gross specimen of CPAM cavity from (a) left upper lobe of lung, (b) right upper lobe of lung, (inset-intact specimen) with cut-open specimen demonstrating cyst CPAM - Congenital pulmonary airway malformation; CECT - Contrast enhanced computed tomography; AP - Antero-posterior; TX - Transverse; SI - Supero-inferior; P - Patient Image Credit: Dr. Vishal V. Bhende

**Figure 6:**
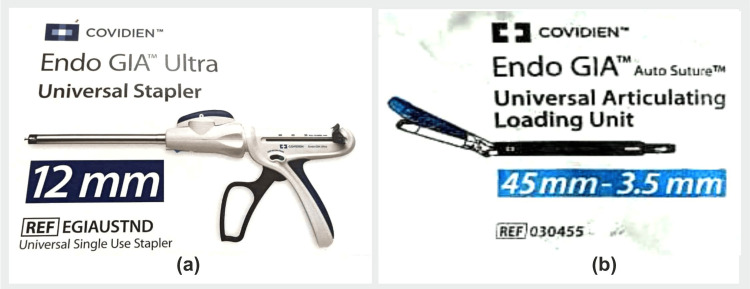
Bronchial stapler provided by Endo-GIA (Covidien, Mansfield, MA, USA) used for closure of right upper lobe bronchus (a) Bronchial stapling gun (b) Cartridges Image Credit: Dr. Vishal V. Bhende

The patient in Case 1 underwent lung-sparing resection (LSR) via left limited lateral thoracotomy. In Case 2, a right upper lobectomy under OLV was performed. The upper lobe bronchial stump was closed by a stapler device using Endo-GIA (Covidien, Mansfield, MA, USA) (Figures 4, 5, 6).

**Figure 7:**
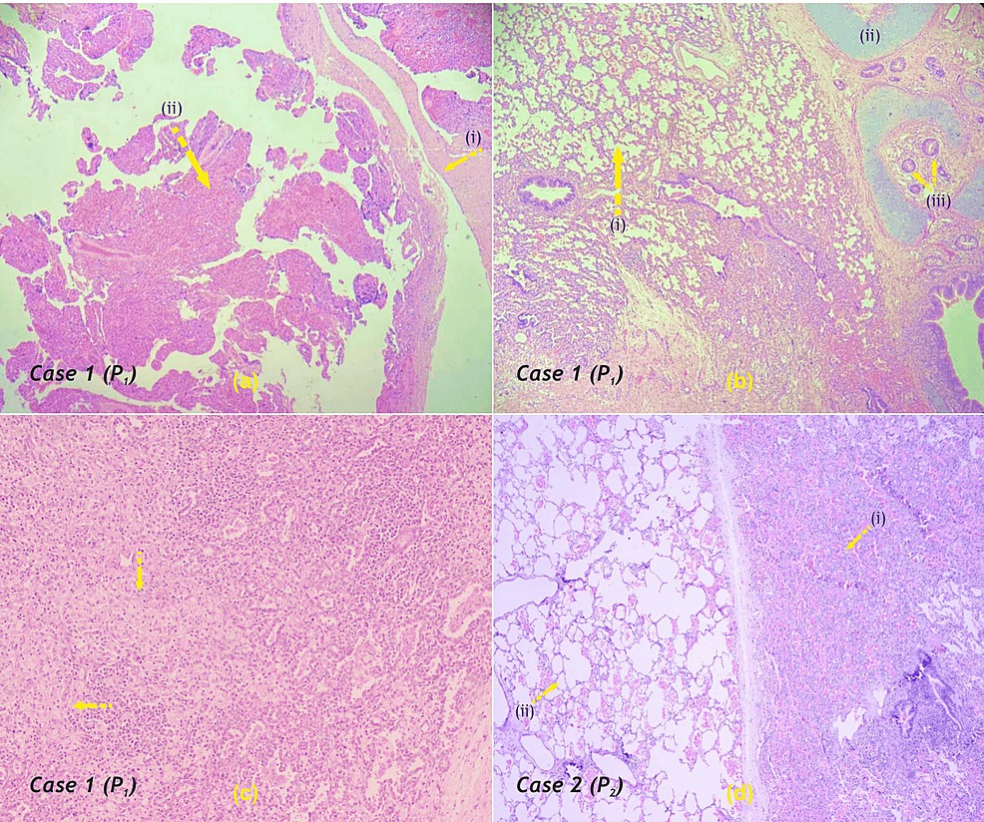
Case 1 (a) Scanner view (4x magnification, H&E stain) showing fibro-collagenous cyst wall, lined by flattened epithelium (i), cyst cavity contains granulation tissue comprised of fibrous tissue, congested blood vessels, inflammatory cells (ii), (b) Low power view (10x magnification) shows adjacent alveolar tissue forming small, irregularly shaped airway spaces (i), lined by ciliated, cuboidal to columnar epithelium. The septa appear thickened and septal spaces appear dilated at places. Immature cartilage (ii) and bronchial glands (iii) are seen within the cyst wall, (c) High power view (40x magnification, H&E stain). Clusters of foamy macrophages and pneumocytes with vacuolated cytoplasm (arrows), Case 2 (d) Right side shows heavily congested lung parenchyma with collapsed alveoli (i), Left side shows adjacent lung tissue with mild congestion and focal alveolar enlargement (ii). H&E - Hematoxylin and Eosin; P - Patient Image Credit: Dr. Zalak N. Parmar

The specimen delivered in both cases were sent for histopathological examination and staining methods were used for demonstration of the cyst wall (Figure 7).

Results

**Figure 8:**
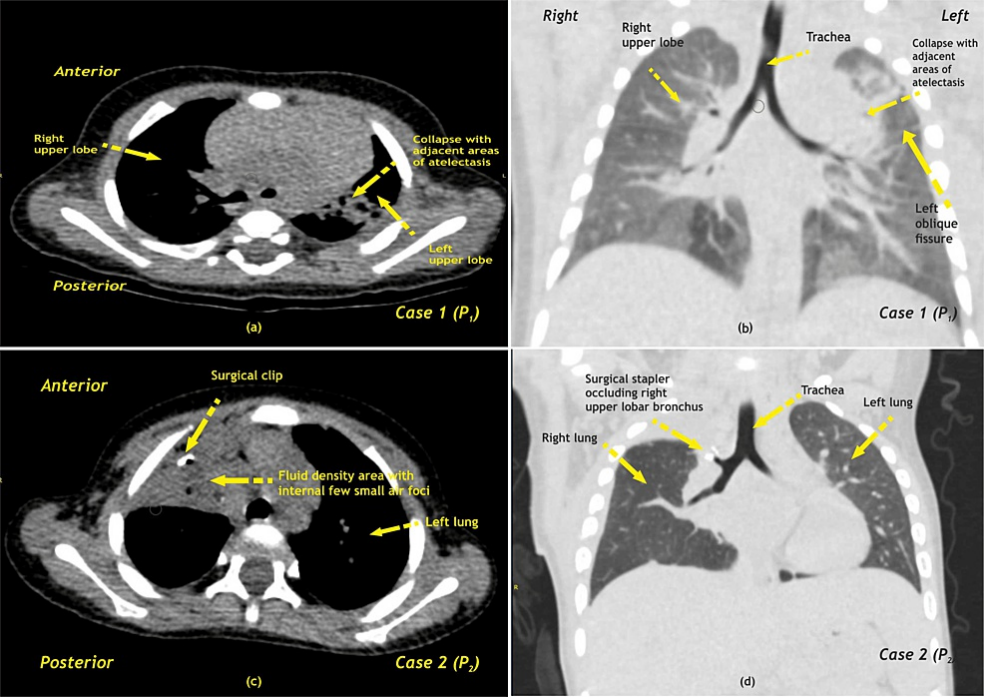
Case 1 (a) HRCT Axial scan chest in mediastinal window, (b) Coronal HRCT scan chest in lung window showing areas of collapse with atelectasis noted in apico-posterior and anterior segments of left upper lobe with mild volume loss of left upper lobe with mild upward displacement of left oblique fissure. Case 2 (c) Postoperative axial HRCT scan of chest in mediastinal window showing mild fluid density areas with internal few small air foci noted in apical and anterior area adjacent to first and second intercostal space right side. There are few internal radiopaque surgical clips noted, (d) Postoperative coronal HRCT scan of chest in lung window showing stapler occluding right upper lobar bronchus. There are areas of adjacent sub-segmental collapse noted. HRCT - High-resolution computed tomography; P - Patient Image Credit: Dr. Dharmendra B. Parmar

Both cases had an uneventful recovery with no complications. A follow-up CECT scan was done at one month post-discharge which revealed no residual lesions (Figure 8, Table 2).

**Table 2:** Surgical Results CPAM - Congenital pulmonary airway malformation; P - Patient; n - Number

Sr. No.	Parameters	Description	Number (n)
1	Type of resection	Non-Anatomical	1
		Lobectomy	1
2	Technique	Primary open Thoracotomy	2
3	Length of hospital stay	12 days (P_1_)	9 days (P_2_)
4	Time of chest tube	3 days (P_1_)	4 days (P_2_)
5	Complications	Nil	Nil
6	Histology	CPAM	CPAM

## Discussion

The range of CLM alternatively known as congenital thoracic malformations, includes diverse conditions like CCAM, congenital lobar emphysema, bronchial atresia with distal cystic degeneration, bronchopulmonary sequestration, bronchogenic and foregut cysts. Although these anomalies may be detected in prenatal assessments, a conclusive diagnosis necessitates histological examination [6,7].

The current treatment scheme indicates that surgical management is essential for alleviating postnatal symptomatic lesions including spontaneous pneumothorax, recurrent infection, hyperinflation, left-to-right shunting, and pulmonary hypoplasia caused by a large mass [8]. Conversely, managing asymptomatic lung lesions is a subject of controversy due to concerns about overall complications and the uncertain long-term outcomes due to a lack of follow-up [9-11].

Advancements in prenatal imaging have heightened the therapeutic challenges in dealing with these infants. The decision-making process is increasingly complex due to the heightened risk of complications associated with CCAM, including respiratory failure, recurrent respiratory tract infections, airway obstruction, and the potential for malignant transformation (bronchioloalveolar carcinoma, rhabdomyosarcoma, pleuropulmonary blastoma) [11,12]. These factors have now become the main criteria for considering surgical resection.

**Figure 9:**
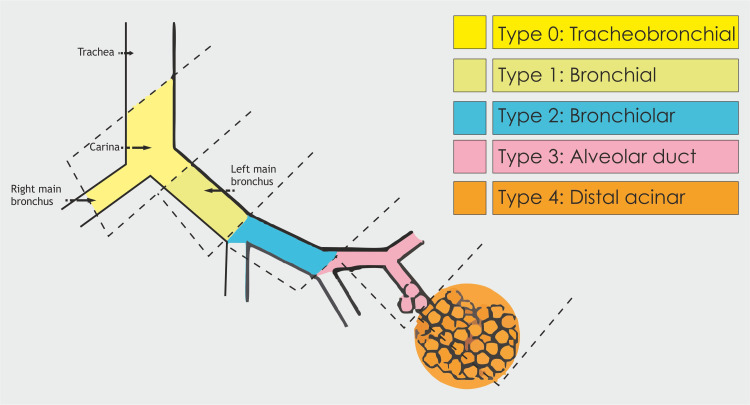
Categorisation of CPAM CPAM - Congenital pulmonary airway malformation Image Credit: Dr. Vishal V. Bhende

CPAM make up 95% of congenital cystic lung diseases. Prenatal screening ultrasound done at 18 - 20 weeks of gestation has shown cystic lung lesion to be the commonly observed anomaly. The documented prevalence of CPAM is roughly 1 per 25,000-35,000 live births [13]. The specific CPAM type reflects the development of abnormalities across the tracheo-bronchial tree (Table 3, Figure 9).

**Table 3:** Types of CPAM CPAM - Congenital pulmonary airway malformation

Sr. No.	CPAM Type	Stocker Classification	Developmental Origin	Proportion of CPAM	Cyst Size	Timing of Presentation	Clinical Presentation
01	Type 0	-	tracheal/bronchial	2%	none	birth	lethal pulmonary hypoplasia
02	Type I	Type I	bronchial/bronchiolar	60-65%	2-10cm	prenatal	asymptomatic, respiratory distress or infection
03	Type II	Type II	bronchiolar	15-25%	0.5-2cm	postnatal	asymptomatic, respiratory distress or infection
04	Type III	Type III	bronchiolar/alveolar	5-10%	microcystic/solid	prenatal	prenatal (hydrops), postnatal respiratory distress
05	Type IV	-	acinar	10%	large multilocular	postnatal	incidental finding

CPAM are typically detected as part of routine prenatal care, leveraging advancements in ultrasound technology. The current state of ultrasound technology provides a heightened sensitivity for the diagnosis of CPAM in the fetus. Additionally, the simultaneous assessment of the cystic pulmonary airway malformation volume ratio (CVR) serves as a sonographic indicator, offering means to evaluate fetuses at risk for hydrops and need for intervention. The CVR represents the mass volume normalized for gestational age and is calculated with the formula for a prolate ellipse [14,15]. The length is determined by the maximal length in sagittal view, while the width and height are measured perpendicular to this axis at the lesion's maximal width. Thus CPAM volume = (Length x Height x Width x 0.52); CVR = (Length x Height x Width x 0.52)/Head Circumference(cm).

**Figure 10:**
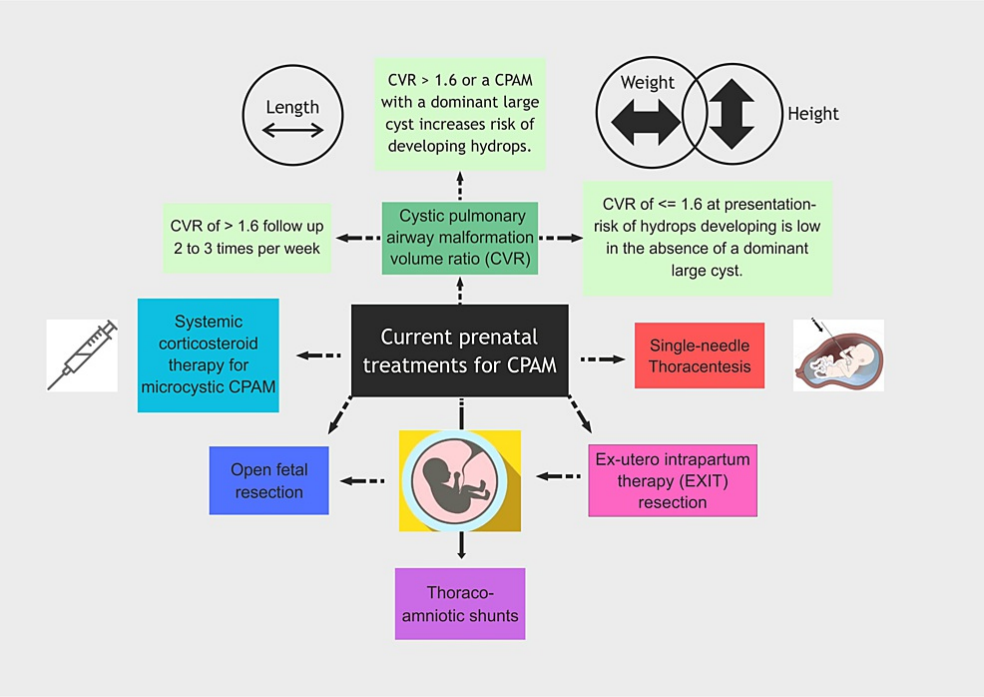
Current prenatal treatments for CPAM CPAM - Congenital pulmonary airway malformation; CVR - Cystic pulmonary airway malformation volume ratio; EXIT - Ex-utero intrapartum therapy Image Credit: Dr. Mathangi Krishnakumar

Fetal therapy has gained popularity owing to its high success rate in the last decade. The current prenatal treatments are described in Figure 10.

Moreover, neonates exhibiting this anomaly typically manifest respiratory difficulties and experience recurrent infectious diseases [16]. The surgical handling of asymptomatic CPAM remains a contentious topic within the realm of pediatric thoracic surgery. Despite numerous investigations, a clear delineation of the optimal timing for surgical intervention and the specific resection procedures has yet to be established.

In our case, patient P1 underwent LSR of the left upper lobe of the lung at two months of age. Concerning the surgical strategy, lobectomy has traditionally been the preferred treatment for CPAM limited to a single lobe. Recently, alternative procedures known as LSR, including segmentectomy and non-anatomic resections like wedge resections, have been suggested [17]. The primary goal of LSR is to retain a significant portion of the lung tissue. Initially applied in cases with multiple lobes affected, certain medical teams have gradually expanded its usage to include a majority of cases, especially for the smallest and most peripheral lesions. In the other case, patient P2 underwent right upper lobectomy at one year, nine months.

While there is widespread agreement on neonatal surgery, concerns have been raised in various reports regarding surgical procedures in infants. The rationale behind advocating surgery for asymptomatic patients with CPAM during infancy lies in the prevention of bacterial dissemination and the potential malignization of cystic wall. It is suggested that asymptomatic infants undergo surgery after reaching one year of age to ensure proper growth [18].

However, conflicting recommendations exist, with some studies proposing earlier surgical interventions than the aforementioned guideline [19,20]. For instance, Style et al. found that patients having surgery at an age less than or equal to four months experienced shorter operative times and anesthesia durations compared to those undergoing late resection (more than four months) [19].

**Figure 11:**
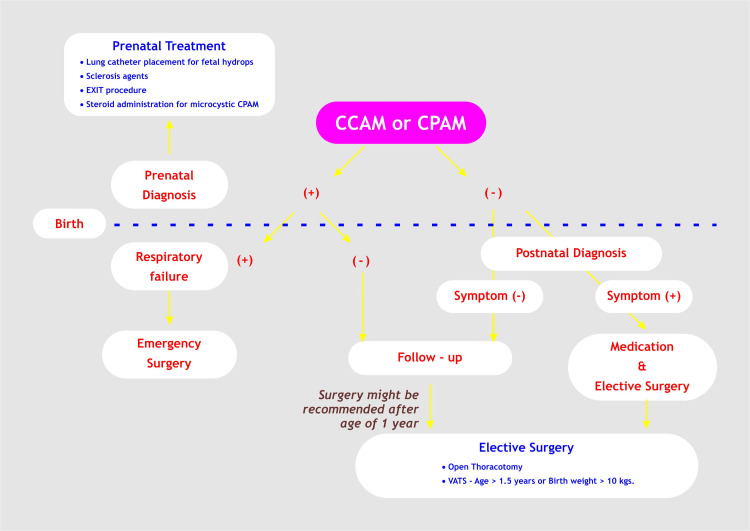
Algorithm of surgical indication for CPAM CCAM - Congenital cystic adenoid malformation; CPAM - Congenital pulmonary airway malformation; EXIT - Ex-utero intrapartum therapy; VATS - Video-assisted thoracic surgery Image Credit: Dr. Vishal V. Bhende

The natural progression of unresected CPAM remains uncertain, as some studies indicate that certain cases may remain asymptomatic for extended periods [9]. Additionally, prior literature has highlighted a paradoxical increase in infections following surgical resection [21]. Figure 11 illustrates the algorithm for surgical indications in CPAM.

## Conclusions

Patients experiencing respiratory failure post-birth due to CPAM necessitate surgery within a week. Despite this urgency, postoperative recovery is generally positive. In our case series, two patients underwent surgery at two months and one year nine months using an open technique. Atypical resection (LSR) emerges as a secure method for preserving lung parenchyma, applicable to both symptomatic and asymptomatic cases. However, there is a need for comparative studies to further validate this technique, as exemplified by its adoption in the P1 patient.

Video-assisted thoracic surgery (VATS) can serve as an initial diagnostic approach in specific centers. Nevertheless, for concealed lesions, the potential for extended operative times with thoracoscopy suggests that an open technique may be more appropriate. Complete video-assisted thoracic surgery (C-VATS) lobectomy becomes a feasible option for those older than 18 months or weighing more than 10 kgs. Given the lower incidence of complications after lung-sparing surgery, we advocate for early elective surgery for these malformations.
